# A functional linguistic analysis of social cohesion impairment in guided interviews conducted with individuals with schizophrenia

**DOI:** 10.1192/j.eurpsy.2023.586

**Published:** 2023-07-19

**Authors:** C. Egyed, R. Herold, A. Hambuch, J. D. Fekete

**Affiliations:** ^1^Department of Languages for Biomedical Purposes and Communication; ^2^Department of Psychiatry and Psychotherapy, University of Pécs Medical School, Pécs, Hungary

## Abstract

**Introduction:**

Individuals with schizophrenia exhibit severe speech and Theory of Mind (ToM) deficits creating substantial handicaps for them on the level of communication and interpersonal skills. Consequently, these individuals cannot adequately take part in social life, and are exposed to marginalization in all aspects of life. Hence, communication impairments associated with schizophrenia are a central issue to investigate in order to optimize their quality of life and functioning in society. The study being part of an interdisciplinary research is based on guided interviews related to a short story by Hemingway. The analysis of person deictic expressions related to social emotions and social interactions combined with the most frequently used mental state terms (e.g. ‘I don’t know’, ‘I think’) identified in the corpus may not only describe the severity of linguistic disturbances indicating ToM deficits but can also help understand patients’ social dysfunction and difficulties in the context of social cohesion.

**Objectives:**

The primary task of the functional linguistic research is to identify and classify the occurrence of linguistic disturbances during mentalizing processes expressed via mental state terms. The study particularly focuses on interpersonal relations expressed with person deictic forms that may indicate the difficulties of this patient group with social cohesion.

**Methods:**

The corpus involves 40 guided interviews including 20 individuals with schizophrenia treated at the Department of Psychiatry of the University of Pécs and 20 controls. The interviews were conducted by a PhD student of Psychology in Hungarian and centred around Hemingway’s short story entitled *The End of Something.* The interviews were digitally recorded and transcribed in Hungarian. The qualitative analysis was performed with Sketch Engine corpus analysis tool, which assisted in the identification and classification of collocations associated with the interviewees’ mental processes directed at interpersonal relations expressed by person deictic forms.

**Results:**

Pragmatic processes including the communicative aspect showed severe deficiencies. The most commonly used mental state term *‘I don’t know*’ combined with person deictic expressions revealed that individuals with schizophrenia have difficulty attributing mental states to a specific linguistic utterance during a social situation (e.g. ‘I don’t know why somebody said that’). These examples show that their communicative and interpersonal skills are substantially impaired.

**Conclusions:**

The findings can offer some possible indications for psychotherapists how to detect pragmatic impairments in schizophrenic speech and interpret mental state terms with reference to social interaction, thereby contributing significantly to therapeutic success enhancing the social reintegration of individuals with schizophrenia.

**Disclosure of Interest:**

None Declared

